# 

**Published:** 1893-12

**Authors:** 


### CONTENTS:

Article I.—The Teeth as an Index of Civilization, By Dr. R. B. Winder. - - - - - - - 337

" II.—Empyema of the Antrum. By A. B. D. Densham. - 339

" III.—Rubber Plates. By T. F. Skeede. - - - 342

" IV.—"The Sixth-Year Molar." By P. P. Sparke, D. D. S. 345

" V.—Development of the Human Tooth. By Dr. E. P. Beadles. - - - - - - - - 351

" VI.—Concerning Various Methods Advocated for Obviating the Necessity of Extracting Devitalized Tooth-Pulps. By Dr. W. D. Miller. - - - - 356

" VII.—Ourselves as Others See Us.—S. S. Towler, M. D. - 364

" VIII.—Hypnotism and Suggestion. - - - - 367

" IX.—Fracture of the Jaw. By Dr. R. Y. Henly. - - 370

### OBITUARY.

In Memory of Dr. Wardlaw, - - - - - - 375

Dr. Geo. W. McElhaney is Dead. - - - - - 377

### EDITORIAL, Etc.

U. S. P. 1890.—The Pharmacopœdia of the United States, Seventh Decennial Revision. - - - - - - - 380

